# Genotyping from targeted NGS data based on a small set of SNPs correctly matches patient samples

**DOI:** 10.1186/s13104-025-07348-3

**Published:** 2025-07-02

**Authors:** Deyan Yordanov Yosifov, Christof Schneider, Stephan Stilgenbauer, Daniel Mertens, Eugen Tausch

**Affiliations:** 1 Division of CLL, Department of Internal Medicine III, Ulm University Hospital, Ulm, Germany; 2 Cooperation Unit “Mechanisms of Leukemogenesis”, German Cancer Research Center (DKFZ), Heidelberg, Germany; 3 Comprehensive Cancer Center Ulm (CCCU), Ulm University Hospital, Ulm, Germany

**Keywords:** Sample misidentification, Sample mislabelling, Sample swaps, Single nucleotide polymorphisms (SNPs), Genotyping, Targeted NGS, R, Maftools

## Abstract

**Objective:**

Mislabelling and swapping of laboratory samples are handling errors that can lead to erroneous interpretation of data and/or patient harm. Sequenced samples can be traced back to the respective donors by matching of single nucleotide polymorphisms (SNPs). Frameworks and software to do this have been developed for use with whole genome/exome sequencing data but not for targeted next-generation sequencing (tNGS), possibly due to the limited genomic coverage with tNGS and the need for individualization of the set of interrogated SNPs. We decided to adapt a popular tool for use with tNGS data, to demonstrate the possibility of selecting informative SNPs from a typical tNGS panel and to create an automated workflow for detection of sample handling errors.

**Results:**

We compiled a custom list of 28 SNPs and with its help we demonstrated the practicability of using only tNGS data to cost-effectively detect mislabelled samples. In two cohorts of totally 1441 patients with sequential samples, we could identify 3 sample swaps, 7 mislabelled samples (3 externally and 4 internally) and 1 mistake of unknown origin. We provide an R function for automated detection of sample swaps and mislabelling to the community as a free and open-source tool.

**Supplementary Information:**

The online version contains supplementary material available at 10.1186/s13104-025-07348-3.

## Background

Inadvertent errors in sample identification such as mislabelling or sample swapping are an important problem in research and clinical laboratory practice that, if not identified early enough, can lead to erroneous interpretation of data and/or patient harm [1–3]. Sample tracking methods and rules for good laboratory practice help to reduce the possibility for occurrence of sample swaps but such events have been repeatedly detected post factum even in large studies by prestigious institutions with established protocols for sample handling [3–5]. While probable misidentification of samples taken for simple biochemical assays would only become suspected if the obtained data do not fit in a logical way with other measured or recorded data, samples taken for genetic/genomic studies can be traced back to the original donor and this can be done proactively as part of the quality control in clinical or research settings. This is especially relevant for big projects with thousands of samples shipped from multiple sample collection sites to a single or several specialized testing centres where they are processed together in big batches [6]. In such setups, sample misidentification can happen at different levels and at different locations along the testing chain, including during transit [1, 2, 7]. The more than thousand samples from chronic lymphocytic leukemia (CLL) patients that we sequence via targeted next-generation sequencing (tNGS) on yearly basis prompted us to establish a procedure for detection of samples with mistaken identity. This can be used both to distinguish samples from different individuals and to confirm that multiple samples from the same person are correctly matched. We envisaged it would be possible to achieve this using only the available tNGS data instead of employing specialized genotyping assays that would increase both costs and labour time.

The two most widely used methods for genetic identification are based on short tandem repeats (STRs) and single nucleotide polymorphisms (SNPs), respectively. STRs are more challenging to work with because they are generally located in non-coding regions and moreover, they are characterized by higher sequencing error rates and often require longer than typical sequencing read lengths to precisely define the number of repeats [4, 7]. This makes them unsuitable for our purpose. In contrast, SNPs are ubiquitous in the genome and simple to assay [4]. Various bioinformatic tools make use of SNPs to detect sample swaps. There are two main types of such programs: (1) tools that compare two binary alignment map (BAM) files by scanning each position and (2) tools that use a predefined list of SNPs and scan only these positions in the BAM files. The first approach is quite slow and as implemented for example in the R package SICtools, it is limited to scanning only a given region, that can at most be a whole chromosome. This is not compatible with typical tNGS panels with genes of interest scattered across different chromosomes; furthermore, results would be influenced by somatic variants in the scanned regions, with possible false mismatches of consecutive tumour samples from the same patient due to clonal evolution. Among the most popular tools that use the second approach with a predefined list of SNPs are the Python program NGSCheckMate [7] and the function sampleSwaps available in the R package maftools [8]. These programs are much quicker but they have been designed to work with whole genome/exome sequencing (WGS/WES) data. Of all 21067 positions in the list of NGSCheckMate, only 5 were covered by our tNGS panel. Of all 6059 positions in the list of maftools (taken from [6]), only 4 were covered by our panel, which would not be sufficient for reliable detection of (mis)matches. These examples show that custom sets of SNPs would be necessary for different tNGS panels. We developed a broadly applicable procedure for definition of such custom sets of SNPs using our CLL tNGS panel as an example, evaluated the performance of the resulting set of SNPs regarding sample mismatch detection and automated the detection process by developing an R function that we provide to the community as a free and open-source tool.

### Approach and SNP selection

**Fig. 1:**
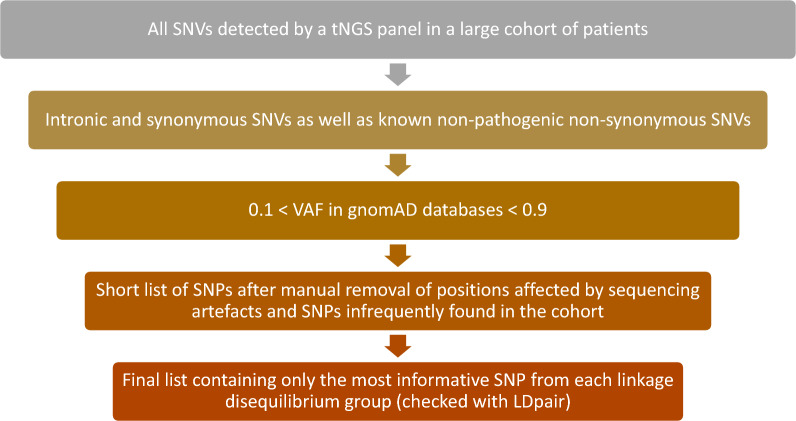
General procedure for selection of SNPs to be used for detection of misidentified samples. SNV—single nucleotide variant; tNGS—targeted next-generation sequencing; SNP—single nucleotide polymorphism

We used the accumulated data from 926 CLL patients sequenced in our laboratory using a custom tNGS panel fully or partially covering the coding regions of 27 genes relevant for CLL pathogenesis, prognosis or treatment outcome prediction (Additional file 1). As a first step, we selected all single nucleotide variants (SNVs) that are normally filtered out by our analysis pipeline (intronic and synonymous variants as well as known non-pathogenic non-synonymous SNPs). Then, similarly to the selection procedure in [6], we selected only those of them with variant allele frequency (VAF) between 0.1 and 0.9 in either the gnomAD genome or gnomAD exome database (because rare or too common SNPs would be of limited value as differentiating markers between samples). We manually reviewed the remaining variants, removing artefacts (“variants” with similar VAF in all samples) and true SNPs found in too few samples (low information contribution). This resulted in a list of 77 SNPs (Additional file 2). Some of these SNPs are located close to each other and thus could be a possible source of bias due to linkage disequilibrium. Therefore, we used the LDpair tool [9] to identify such correlated SNPs and from each linkage group we chose the SNP whose VAF was closest to 0.5 to maximize the information content. This procedure resulted in a final list of 28 SNPs (11 located in introns, 1 in a polypyrimidine tract, 2 in 5’-UTR, 2 in 3’-UTR and 12 in exons; Table 1). The general procedure for SNP selection is summarized in Fig. 1.

**Table 1:** List of SNPs chosen for genotyping, separated according to their genomic location and functional consequences

Intronic	Polypyrimidine tract	5’-UTR	3’-UTR	Exonic (synonymous)	Exonic (missense)
rs969273	rs12446127	rs16837903	rs12644477	rs724710	rs2286615
rs2233437		rs1642785	rs1055088	rs2229974	rs5759408
rs10263573				rs1143685	rs1985791
rs664982				rs1143686	rs2009433
rs76433096				rs1143689	
rs12445580				rs1071644	
rs11865395				rs1801018	
rs4889430				rs62218846	
rs1805419					
rs738094					
rs3747288

### Implementation

We decided to base our software implementation on the sampleSwaps function from the R package maftools. By default, this function uses the list of SNPs published by Westphal et al. [6]. We tweaked it to accept alternative lists and introduced additional modifications into its code: we made it to return not only the number of discordant SNPs but also their positions, fixed a bug that resulted in a fatal error when there were no discordant SNPs between any two samples and reduced the threshold of concordant SNPs from 80 to 70%. The latter change led to more reliable detection of matched samples in our testing (see below).

When analysing a batch of samples for possible mismatches, it is important not only to identify all matching samples but also to check whether samples that should match, such as multiple samples from the same subject, do indeed match. To make such a thorough analysis easier to perform, we wrote a wrapper function that: (1) uses the modified sampleSwaps function with a custom list of SNPs to identify all matching samples in a batch of samples; (2) outputs a CSV file named Pairwise_concordance_[suffix]_[time_stamp].csv that contains the fraction of concordant SNPs and the correlation coefficient for each possible pair of samples (examples in Additional files 3 and 4); (3) outputs a CSV file named SNP_readcounts_[suffix]_[time_stamp].csv that contains the number of reads from the reference and alternative alleles, and the VAF of each SNP in each sample (example in Additional file 5); (4) optionally outputs a CSV file named Expected_concordant_pairs_[suffix]_[time_stamp].csv that includes all expected pairs of samples, with calculated fraction of concordant SNPs and correlation coefficient for each pair (examples in Additional files 6 and 7); (5) optionally produces a PNG file named Pairwise_concordance_[suffix]_[time_stamp].png: a heatmap of the correlation coefficients for all possible pairs of samples. The concordant pairs (if any) appear at the top of the table in Pairwise_concordance_[suffix]_[time_stamp].csv. All expected concordant pairs, whether really concordant or not, are listed in Expected_concordant_pairs_[suffix]_[time stamp].csv in order of descending correlation coefficients. Our R function and the list of SNPs are available in Additional file 8 and also at GitHub: https://github.com/deyanyosifov/swap_checker.

### Validation

We used our function with the BAM files (coordinate-sorted and indexed) obtained after sequencing the 926 samples that served as the basis for SNP selection (timepoint 1; TP1) and follow-up samples from 228 of the patients (TP2), 1154 BAM files in total. The analysis took about 3 h computation time on a server with 16 Intel® Xeon^®^ Platinum 8260 (2.4 GHz) CPUs and 128 GB of random-access memory (RAM), and identified 231 tentative pairs of samples (Additional file 3). Most of these were the expected pairs of TP1 and TP2 samples, however there were also 6 unexpected pairs consisting of samples labelled with discordant patient identification numbers (Table 2; rows highlighted in red in Additional file 3). Conversely, 5 of the 229 expected pairs had discordant SNP profiles (Table 3; rows highlighted in red in Additional file 6). The identified mismatches were traced back to 4 original mistakes: one sample mislabelling explaining one unexpected pair and the lack of concordance in one expected pair; and 3 sample swaps explaining 5 of the unexpected pairs and the lack of concordance in 4 expected pairs. The swapping errors could be independently validated by comparing the IGHV-hypermutation status and fluorescent in situ hybridization (FISH) profiles of the affected samples. Details are provided in Additional file 9.

**Table 2:** Unexpected pairing of samples from 926 CLL patients

Unexpected pair	X sample	Y sample	Fraction of concordant SNPs	Correlation coefficient
1	CLL-68035556-02S03659-TP1	CLL-68035557-67S38544-TP2	0.857	0.999
2	CLL-2715509-04S0752-TP1	CLL-07925505-66S28936-TP2	1	1
3	CLL-02225553-04S3522-TP1	CLL-08865502-04S00318-TP1	1	1
4	CLL-08865502-60S06293-TP2	CLL-02225553-04S00319-TP3*	0.821	0.999
5	CLL-8095559-04S00113-TP1	CLL-625556-04S00065-TP2	0.929	0.999
6	CLL-625556-04S00065-TP2	CLL-8095559-60S7426-TP2	0.893	0.998

The first set of digits in each sample name is the patient identification number. TP1—timepoint 1; TP2—timepoint 2

*This sample was sequenced additionally to help us correctly assign the swapped samples from unexpected pair 3

**Table 3:** Expected pairs that were actually not concordant

Expected pair^#^	X sample	Y sample	Fraction of concordant SNPs	Correlation coefficient
1	CLL-68035557-02S65286-TP1	CLL-68035557-67S38544-TP2	0.321	0.241
2	CLL-07925505-04S0753-TP1	CLL-07925505-66S28936-TP2	0.286	0.19
3	CLL-02225553-04S3522-TP1	CLL-02225553-04S00319-TP3*	0.429	0.266
4	CLL-08865502-04S00318-TP1	CLL-08865502-60S06293-TP2	0.357	0.279
5	CLL-625556-02S03235-TP1	CLL-625556-04S00065-TP2	0.357	0.449

The first set of digits in each sample name is the patient identification number. TP1—timepoint 1; TP2—timepoint 2

*This sample was sequenced additionally to help us correctly assign the swapped samples from unexpected pair 3. ^#^ Only the expected pairs that did not really match are shown. All expected pairs are available in Additional file 6

Additionally, we identified 46 borderline cases with high correlation coefficients (≥ 0.9 as specified by the sampleSwaps function) but with lower fraction of concordant SNPs (< 0.8; both thresholds need to be satisfied for the function to flag samples as potentially paired). Manual analysis of these cases using the information about the VAF of each SNP (Additional file 5) revealed that the lower fraction of concordant SNPs was in most cases due to few SNPs with differing VAFs, mostly on the same chromosome, that could be easily explained by the presence of clones with chromosome aberrations that changed in size over time. This hypothesis was confirmed by comparison with FISH data. All remaining cases could be explained by stochastic effects due to low sequencing coverage of some of the SNPs in one of the paired samples or by differences in the cancer cell fraction. Overall, as pairs with fractions of concordant SNPs between 0.7 and 0.8 were numerous (31) but had only minimal deviations of VAF, we decided to reduce the threshold to 0.7 in the modified sampleSwaps function (see above) and in the future to manually inspect only pairs below this threshold (rows highlighted in yellow in Additional file 6).

In addition, we analysed possible sample mix-ups in another independent patient cohort (n = 515) sequenced with the same tNGS panel, where we identified 7 cases of mislabelled samples (3 external, 3 internal and 1 of unclear origin), as detailed in Additional file 9.

Finally, we analysed how the sensitivity and specificity of the approach would be decreased if a lower number of SNPs were used. To this end, we repeated the analysis of the first cohort with randomly chosen subsets of SNPs from the original list (10 iterations for each of the chosen subset sizes—26, 24, 22, 20, 18 and 16 SNPs). Even as few as 18 SNPs were enough to detect all 6 unexpected pairs and all 5 discordant pairs in each of the 10 iterations (maintained high sensitivity) but reducing the number of SNPs led to an exponential increase of the number of false positives (reduced specificity; Additional file 10). Based on these results, it can be recommended that custom assays should cover at least 25 independent SNPs usable for genotyping.

### Limitations

Our work demonstrates the practicability of using only tNGS data to cost-effectively detect sample swaps and other sources of sample misidentification. For this, we had to create a custom list of SNPs based on the genes covered by our sequencing panel but we believe our approach for selection of SNPs could be used by other researchers to similarly create custom lists for their respective gene panels.

It has been shown that 30 to 80 statistically independent SNP positions are enough to uniquely identify a single person, depending on the heterozygosity of the SNPs and their frequency variation among different ethnic groups [10, 11]. Typical forensic-grade SNP panels for individual identification contain 45 to 52 SNPs [12–14]. Our list contains 28 SNPs, which might not be enough to unequivocally identify each person on Earth but would be sufficient to uniquely and with high confidence identify each subject in a large clinical trial with thousands of participants. In the two patient cohorts that we analysed (n = 1441), we could identify 3 sample swaps, 7 mislabelled samples (3 externally and 4 internally) and 1 case in which the source of an apparent sample misidentification could not be found. This high rate of detection was possible because a lot of the patients provided consecutive samples for monitoring their disease, which allowed us to detect not only unexpectedly matching samples but also cases in which the expected matching between consecutive samples from the “same” patient was absent. This also allowed us to deduce exactly how sample swaps happened and which samples are misidentified, and to re-link the sequencing data with the true patient of origin in the majority of problem cases. An analysis of a cohort of patients that provided samples at a single timepoint can still be worth as it would identify instances in which a sample has been sequenced twice under two different names (similarly to the case with the externally mislabelled samples in our second validation study; also possible in scenarios when a single patient visits two or more hospitals, all of which send pseudonymized samples to a central laboratory) but a sample swap or a simple sample mislabelling would remain undetected. Another potential benefit could be detection of samples with small contamination from another sample, as in this case the VAFs of some of the SNPs would deviate from the usual distribution pattern (0, 0.5 and 1) and it could even be possible to identify the source of the contaminating DNA.

The function we provide will help anyone with basic knowledge of R to detect sample swaps and mislabelling from aligned sequencing data (BAM files). A prerequisite is the selection of an appropriate set of SNPs both in terms of quantity (at least 25 and up to 80 if possible—more is unnecessary and would just increase computational burden) and quality (highly variable in the population of interest and not correlated due to linkage disequilibrium). In this process, one should consider the ethnic diversity of the target population. If broad applicability is desired, SNPs should be chosen that are highly variable in all ethnic groups, however this additional restriction might result in too few usable SNPs. The other approach is to tailor the SNP list according to the target population and if this is a specific ethnic group one can use the respective allele frequencies for that group from gnomAD for filtering (VAF in the range 0.1–0.9), or one can use data from own research on a representative sample of the target population, as we did.

Users could execute the function after each sequencing run to detect handling errors within the current batch of samples and/or after all samples belonging to a large study have been sequenced to detect more obscure sample swaps. It should be noted that processing time can become very long with studies of more than 5000 samples due to the large number of possible combinations to be tested.

## Supplementary Information

Additional file 1. BED file. tNGS panel design covering fully or partially the coding regions of 27 genes relevant for CLL pathogenesis, prognosis and/or prediction of treatment outcomes.

Additional file 2. BED file. List of 77 informative SNPs found in the genomic regions covered by the sequencing panel (Additional file 1).

Additional file 3. Excel table (XLSX file). Pairwise concordance of samples from the first analysed cohort of patients (n=926). The table was originally generated by the swap_checker() function in CSV format and then Excel was used to highlight unexpected pairs.

Additional file 4. Excel table (XLSX file). Pairwise concordance of samples from the second analysed cohort of patients (n=515). The table was originally generated by the swap_checker() function in CSV format and then Excel was used to highlight unexpected pairs.

Additional file 5. Excel table (XLSX file). Number of reads from the reference and alternative allele and the VAF of each SNP in each sample of the first analysed cohort of patients (n=926). The table was originally generated by the swap_checker() function in CSV format and then resaved in Excel format.

Additional file 6. Excel table (XLSX file). Expected concordant pairs of samples in the first analysed cohort of patients (n=926). The table was originally generated by the swap_checker() function in CSV format and then Excel was used to highlight discordant pairs (red) or pairs with a fraction of concordant SNPs below 70% (yellow).

Additional file 7. Excel table (XLSX file). Expected concordant pairs of samples in the second analysed cohort of patients (n=515). The table was originally generated by the swap_checker() function in CSV format and then Excel was used to highlight discordant pairs (red) or pairs with a fraction of concordant SNPs below 70% (yellow).

Additional file 8. ZIP file. This archive file contains the Swap_checker.R file, which includes the wrapper swap_checker() function, with built-in modified sampleSwaps() function and the list of 28 SNPs used for detection of swapped samples from CLL patients. The archive also includes the list of SNPs as a separate BED file (SNPs_selected.bed; not necessary for using the function but useful as a template for creating custom lists of SNPs) and a help file (README.md).

Additional file 9. Word document (DOCX file). Detailed description of the mistakes found in the two validation cohorts.

Additional file 10. PowerPoint slide (PPTX file). Sensitivity and specificity analysis with respect to the number of SNPs that are used for detection of improperly paired samples – matching unexpected pairs (above) or not matching expected pairs (below). The samples from the first cohort were compared using randomly chosen subsets of SNPs from the original list (10 iterations for each of the following subset sizes – 26, 24, 22, 20, 18 and 16 SNPs). Even as few as 18 SNPs were enough to detect all 6 unexpected pairs and all 5 discordant pairs in each of the 10 iterations. With 16 SNPs, the 6 unexpected pairs could be detected in 8 of 10 iterations, while in 2 iterations only 5 unexpected pairs could be detected (difficult to see on the graph).

## Data Availability

The swap_checker function, which includes the modified sampleSwaps function and our SNP list, is available in Additional file 8, as well as at GitHub (https://github.com/deyanyosifov/swap_checker). The swap_checker function is written in R and is platform independent. It is provided under the GPL-3 license for all users. Requirements: working installation of R (version 4.2 or higher) with the package maftools. Sequencing data used in this study have been deposited at the European Genome-phenome Archive (EGA) under accession number EGAS50000000532.

## Abbreviations

CLL: Chronic lymphocytic leukemia
tNGS: Targeted next-generation sequencing
STR: Short tandem repeat
SNP: Single nucleotide polymorphism
SNV: Single nucleotide variant
BAM: Binary alignment map
WGS: Whole genome sequencing
WES: Whole exome sequencing
VAF: Variant allele frequency
FISH: Fluorescent in situ hybridization
IGHV: Immunoglobulin heavy chain variable region
CPU: Central processing unit
RAM: Random-access memory
