# Supplementary material for: Genotyping from targeted NGS data based on a small set of SNPs correctly matches patient samples
Source: BMC Res Notes. 2025 Jul 2;18:270. doi: 10.1186/s13104-025-07348-3 (PMC12225085; doi:10.1186/s13104-025-07348-3)
Supplement: Supplementary file 9 — Additional file 9. Word document (DOCX file). Detailed description of the mistakes found in the two validation cohorts. [file 13104_2025_7348_MOESM9_ESM.docx]

**Detailed analysis of sample (mis)pairings in Tables 2 and 3**

The observation that the TP1 and TP2 samples of patient 68035557 did not match (expected pair 1) whereas the TP2 sample of patient 68035557 unexpectedly matched the TP1 sample of patient 68035556 (unexpected pair 1) hinted that the TP2 sample actually originated from patient 68035556. This error was found to be due to mistyping the sample name in the NGS sample sheet (Supplementary Figure 1). Similarly, the TP1 and TP2 samples of patient 07925505 did not match (expected pair 2) whereas the TP2 sample of patient 07925505 unexpectedly matched the TP1 sample of patient 2715509 (unexpected pair 2). However, detailed investigation revealed that here the error was more complex and was due to swapped samples (04S0752 and 04S0753) that were collected on the same date, which led to wrong association of sample ID with patient number. Consequently, not only the sequencing data but also the fluorescent in situ hybridization (FISH) and IGHV-hypermutation status results for these two patients were found to be swapped (independent validation). Analogous was the situation with samples 04S00318 and 04S00319. This sample swap remained initially unresolved as sample 04S00319 was not part of our initial sample collection, but it was sequenced afterwards. This single error explains unexpected pairs 3 and 4 and the absence of concordance in expected pairs 3 and 4. An analogous swapping error occurred also with samples 04S00065 and 04S00060 (not included in the initial sample collection), explaining unexpected pairs 5 and 6 and the absence of concordance in expected pair 5.

| 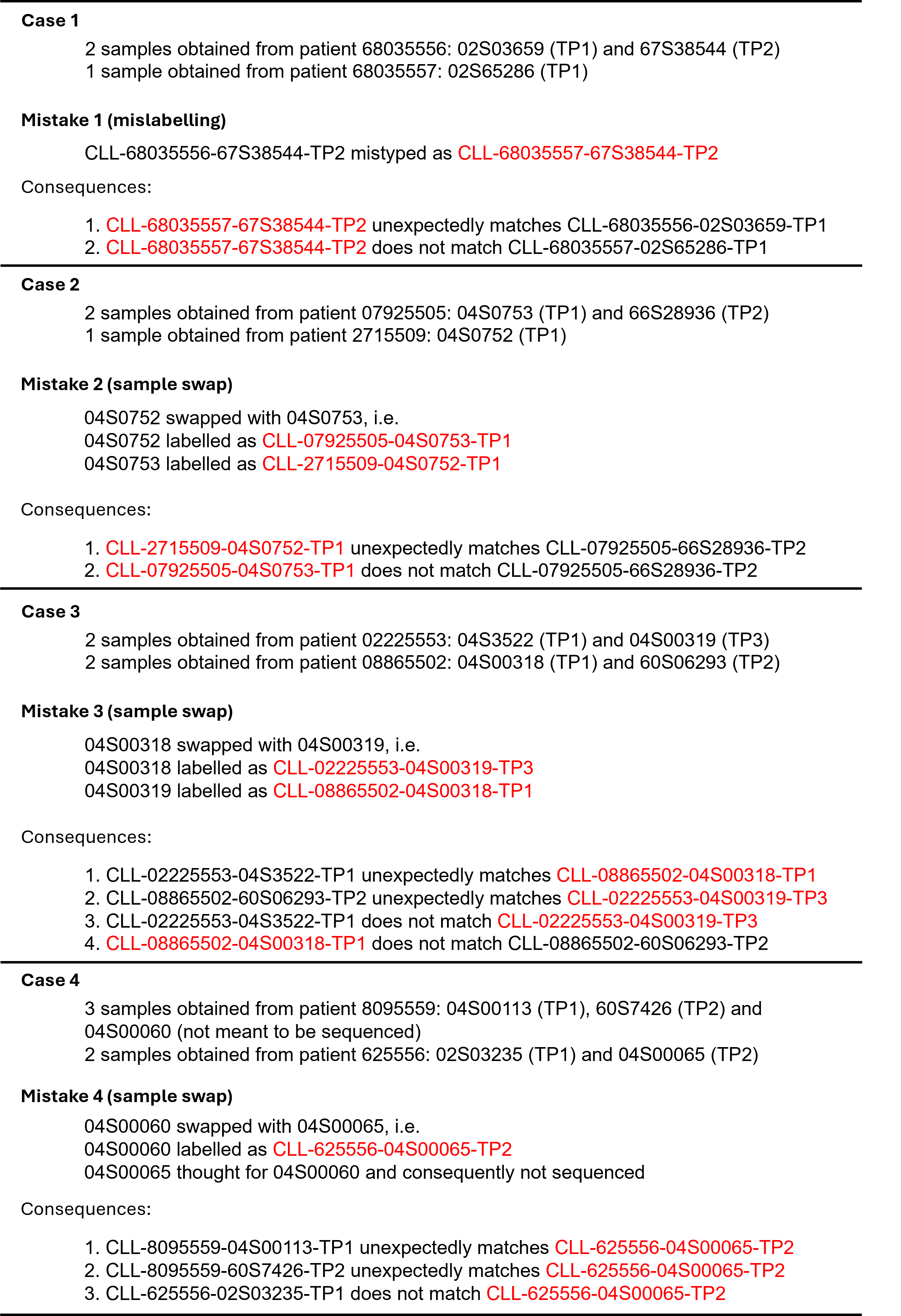 |
| --- |
| **Supplementary Figure 1.** Mistakes identified in the first patient cohort. Mislabelled samples are marked in red. TP1 – timepoint 1; TP2 – timepoint 2. |

**Validation in an independent collection of samples**

We analysed possible sample mix-ups in another cohort of CLL patients (n=515) sequenced with the same tNGS panel as in the first validation. Most of the patients had provided two or more consecutive samples. The analysis took about 90 minutes computation time and identified 453 tentative pairs of samples (Additional file 4). Most of these were the expected pairs of longitudinal samples from the same patient, however there were also 9 unexpected pairs consisting of samples from different patients (Supplementary Table 1; rows highlighted in red in Additional file 4). Conversely, 4 of the expected pairs were not really concordant (Supplementary Table 2; rows highlighted in red in Additional file 7).

**Supplementary Table 1. Unexpected pairing of samples from 515 CLL patients.**

| **Unexpected pair** | **X sample** | **Y sample** | **Fraction of concordant SNPs** | **Correlation coefficient** |
| --- | --- | --- | --- | --- |
| 1 | CLL-079319-02S08063-TP1 | CLL-071129-66S0290-TP2 | 0.929 | 0.999 |
| 2 | CLL-076777-02S9328-TP1 | CLL-077969-02S2398-TP2 | 0.929 | 0.999 |
| 3 | CLL-003221-01S5058-TP1 | CLL-066394-65S0963-TP2 | 0.75 | 0.998 |
| 4 | CLL-057999-09S5471-TP1 | CLL-050143-08S60608-TP1 | 1 | 0.999 |
| 5 | CLL-050143-08S60608-TP1 | CLL-057999-66S7572-TP2 | 0.893 | 0.999 |
| 6 | CLL-057382-09S0064-TP1 | CLL-2249-09S0876-TP1 | 0.786 | 0.996 |
| 7 | CLL-2249-09S0876-TP1 | CLL-057382-66S1562-TP2 | 0.929 | 0.976 |
| 8 | CLL-055709-08S04321-TP1 | CLL-006561-66S8223-TP2 | 0.857 | 0.999 |
| 9 | CLL-055709-66S8223-TP2 | CLL-006561-66S8223-TP2 | 0.929 | 0.925 |
| TP1 – timepoint 1; TP2 – timepoint 2. | | | | |

**Supplementary Table 2. Expected pairs in the second cohort that were actually not concordant.**

| **Expected pair** | **X sample** | **Y sample** | **Fraction of concordant SNPs** | **Correlation coefficient** |
| --- | --- | --- | --- | --- |
| 1 | CLL-2249-09S0876-TP1 | CLL-2249-09S06459-TP2 | 0.5 | 0.529 |
| 2 | CLL-006561-03S60998-TP2 | CLL-006561-66S8223-TP2 | 0.393 | 0.247 |
| 3 | CLL-006561-09S00705-TP1 | CLL-006561-66S8223-TP2 | 0.393 | 0.25 |
| 4 | CLL-40489-08S01897-TP1 | CLL-40489-66S1392-TP2 | 0.393 | 0.014 |
| Only the expected pairs that did not really match are shown. All expected pairs are available in Additional file 7. TP1 – timepoint 1; TP2 – timepoint 2. | | | | |

Unexpected pairs 1, 2 and 3 were found to be due to mislabelling at the external institutions that sent these samples to us for analysis; in reality, the samples in the left column were from patients 071129, 077969 and 066394, respectively (Supplementary Figure 2). The observation that sample CLL-050143-08S60608-TP1 correlates with both the TP1 and TP2 samples of patient 057999 (unexpected pairs 4 and 5), which two samples correlate as expected, hints that either CLL-050143-08S60608-TP1 is a mislabelled sample from patient 057999 or that an operator error resulted in a sample from patient 057999 being sequenced in place of the intended sample. Similarly, unexpected pairs 6 and 7 and the lack of concordance in expected pair 1 can be explained by an analogous mistake affecting sample CLL-2249-09S0876-TP1. Unexpected pairs 8 and 9 and the lack of concordance in expected pairs 2 and 3 can all be explained by a single labelling error, namely CLL-006561-66S8223-TP2 is actually identical with CLL-055709-66S8223-TP2. The true TP2 sample of patient 006561 was sequenced under the name CLL-006561-03S60998-TP2 and was concordant with the respective TP1 sample, as expected. The lack of concordance in expected pair 4 indicates that one of its samples must have been mislabelled (not clear which, as at this stage none of them matches other samples).

| 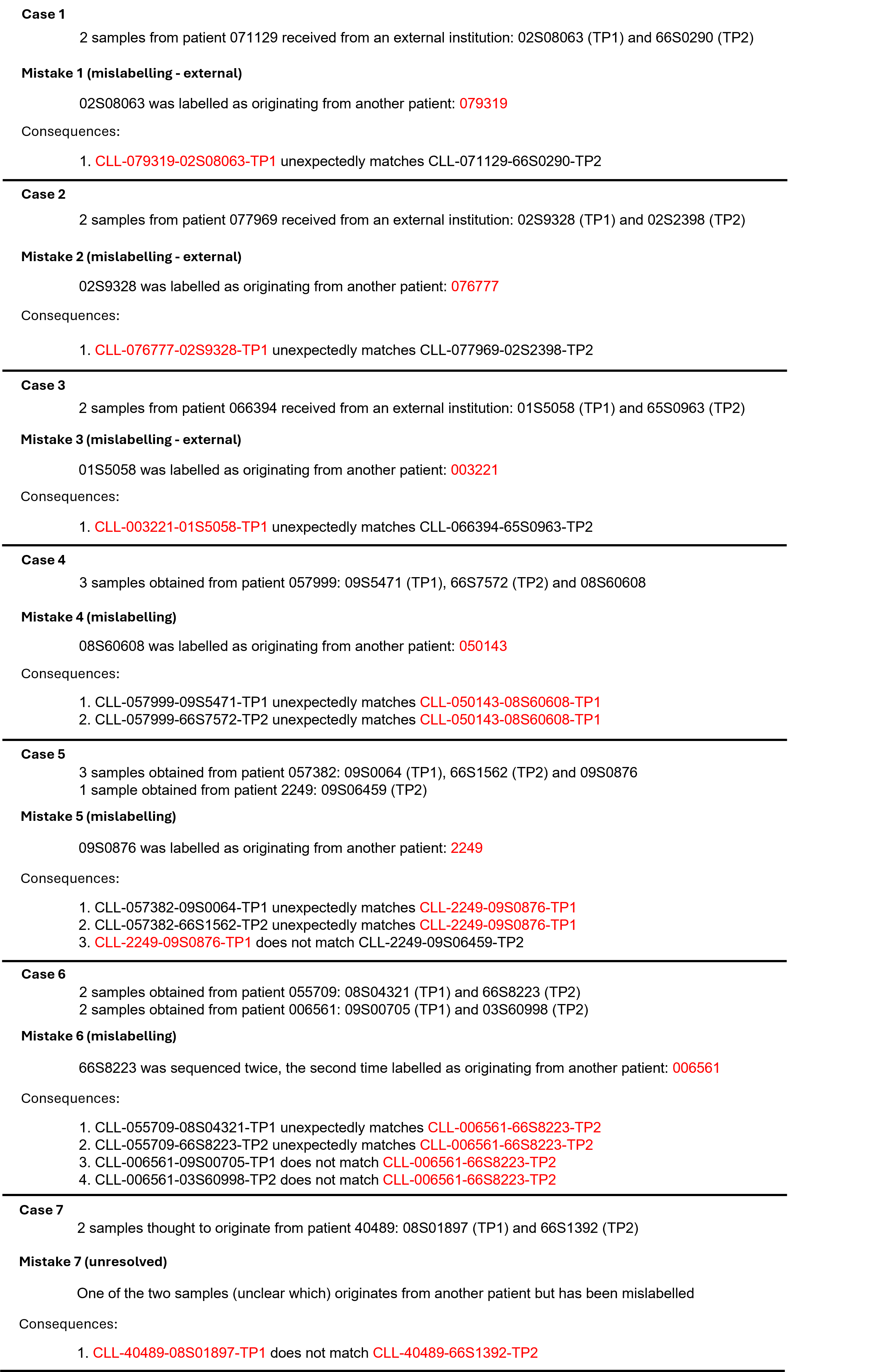 |
| --- |
| **Supplementary Figure 2.** Mistakes identified in the second patient cohort. Mislabelled or probably mislabelled samples are marked in red. |

As in the first analysed cohort, we identified 15 borderline cases with high correlation coefficients (≥0.9) but lower fractions of concordant SNPs (<0.7); the reasons for that were the same as in the analysis of the first cohort and no sample misidentification was involved.

In summary, the analysis of this cohort identified 7 cases of mislabelled samples (3 external, 3 internal and 1 of unclear origin).
