# Supplementary figures and images for: Genotyping from targeted NGS data based on a small set of SNPs correctly matches patient samples
Source: BMC Res Notes. 2025 Jul 2;18:270. doi: 10.1186/s13104-025-07348-3 (PMC12225085; doi:10.1186/s13104-025-07348-3)

## Slide 1
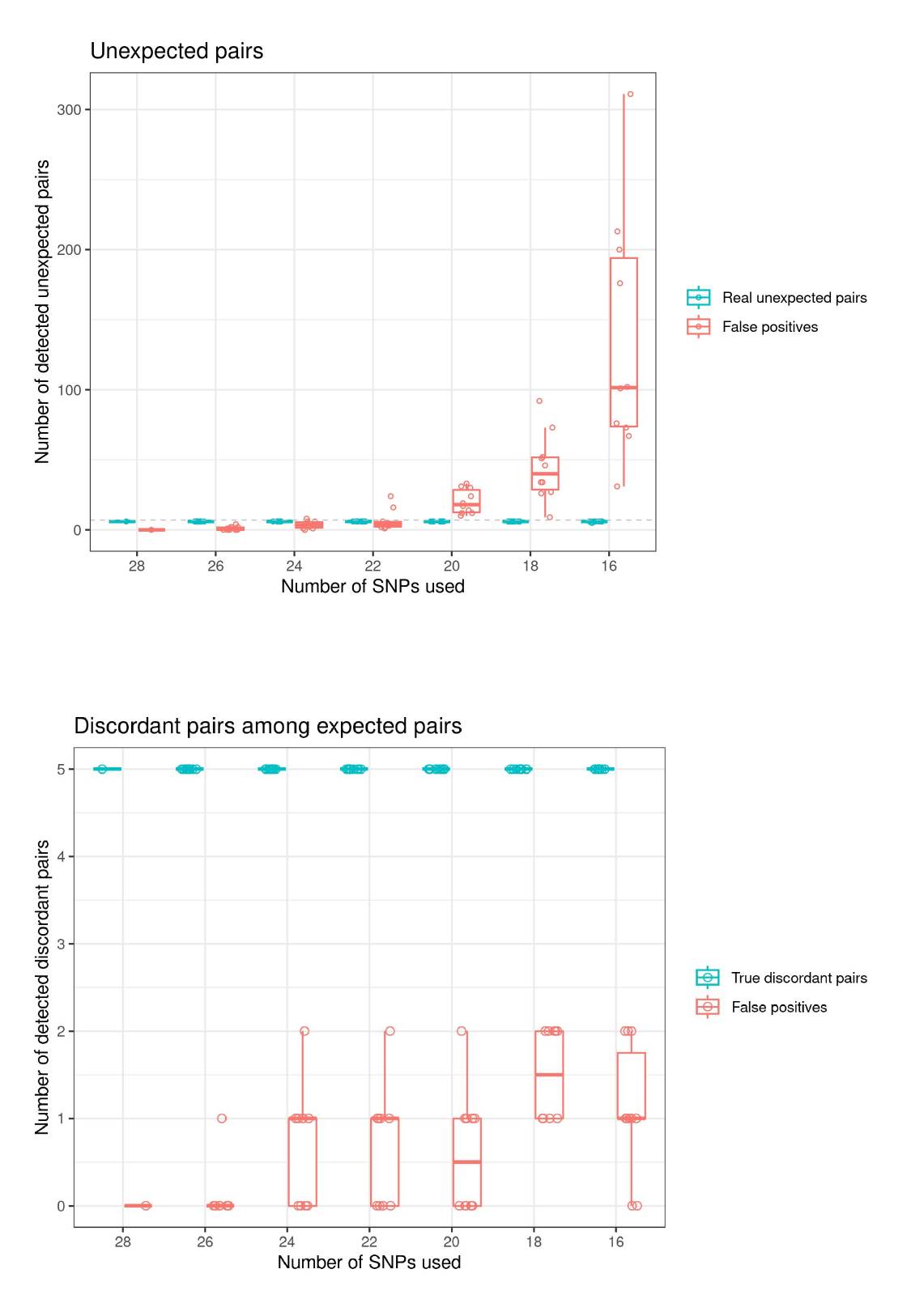

Supplement: Supplementary file 10 — Additional file 10. PowerPoint slide (PPTX file). Sensitivity and specificity analysis with respect to the number of SNPs that are used for detection of improperly paired samples – matching unexpected pairs (above) or not matching expected pairs (below). The samples from the first cohort were compared using randomly chosen subsets of SNPs from the original list (10 iterations for each of the following subset sizes – 26, 24, 22, 20, 18 and 16 SNPs). Even as few as 18 SNPs were enough to detect all 6 unexpected pairs and all 5 discordant pairs in each of the 10 iterations. With 16 SNPs, the 6 unexpected pairs could be detected in 8 of 10 iterations, while in 2 iterations only 5 unexpected pairs could be detected (difficult to see on the graph). [file 13104_2025_7348_MOESM10_ESM.pptx]
